# Parasitostatic effect of maslinic acid. II. Survival increase and immune protection in lethal *Plasmodium yoelii*-infected mice

**DOI:** 10.1186/1475-2875-10-103

**Published:** 2011-04-25

**Authors:** Carlos Moneriz, Patricia Marín-García, José M Bautista, Amalia Diez, Antonio Puyet

**Affiliations:** 1 Departamento de Bioquímica y Biología Molecular IV, Universidad Complutense de Madrid, Facultad de Veterinaria, E28040 Madrid, Spain; 2 Departamento de Ciencias Morfológicas y Biomedicina, Facultad de Ciencias Biomédicas, Universidad Europea de Madrid, 28640, Madrid, Spain; 3 Instituto de Investigación Hospital 12 de Octubre, Universidad Complutense de Madrid, E28040 Madrid, Spain; 4 Departamento de Bioquímica, Facultad de Medicina, Universidad de Cartagena, Cartagena, Colombia

## Abstract

**Background:**

The anti-malarial activity of maslinic acid (MA), a natural triterpene which has been previously shown to exert a parasitostatic action on *Plasmodium falciparum *cultures, was analysed *in vivo *by using the *Plasmodium yoelii *17XL murine model.

**Methods:**

ICR mice were infected with *P. yoelii *and treated with a single dose of MA by a intraperitoneal injection of MA (40 mg kg^-1 ^day^-1^) followed by identical dose administration for the following three days. Parasitaemia and accumulation of intraerythrocytic stages was monitored microscopically. To assess protective immunity, cured mice were challenged with the same dose of parasites 40 days after recovery from the primary infection and parasitaemia was further monitored for 30 days. Humoral response was tested by ELISA and visualization of specific anti-*P. yoelii *antibodies was performed by Western-blotting.

**Results:**

ICR mice treated with MA increased the survival rate from 20% to 80%, showing an arrest of parasite maturation from day 3 to 7 after infection and leading to synchronization of the intraerythrocytic cycle and accumulation of schizonts by day 6, proving that MA also behaves as a parasitostatic agent *in vivo*. Mice which survived the primary infection displayed lower rates of parasitic growth, showing a decline of parasitaemia after day 15, and complete clearance at day 20. These mice remained immunoprotected, showing not malaria symptoms or detectable parasitaemia after rechallenge with the same lethal strain. The analysis of specific antibodies against *P. yoelii*, present in mice which survived the infection, showed a significant increase in the number and intensity of immunoreactive proteins, suggesting that the protected mice may trigger a strong humoral response.

**Conclusion:**

The survival increase observed in MA-treated mice can be explained considering that the parasitostatic effect exerted by this compound during the first days of infection increases the chances to develop effective innate and/or acquired immune responses. MA may represent a new class of anti-malarial compounds which, as a consequence of its parasitostatic action, favours the development of more effective sterilizing immune responses.

## Background

Despite the extensive research carried out to find new anti-malarial drugs or antigenic targets which can be eventually used in vaccine formulations, most of these efforts have been unsuccessful so far due to several factors, such as the emergence of resistant strains, the requirement of both low-toxicity and low-cost anti-malarials, and the failure to develop a practical vaccine that prevents malaria.

There is increasing evidence that the *in vivo *response to anti-malarial treatments is affected by factors other than the intrinsic susceptibility of *Plasmodium *species to the drugs. The parasite load, innate host resistance to the parasite [1] and naturally acquired immunity are known to play an important role in the infection progress and the outcome of the treatment with anti-malarials (reviewed by [2,3]). While most evidence on this subject was gathered from epidemiological surveys in human populations, few experimental studies have been performed using animal models to reproduce the influence of anti-malarial drug-treatments on the onset of acquired immunity. In a recent report, immunization of BALB/c mice with live *Plasmodium yoelii *nonlethal strains under curative chloroquine doses showed to confer protection against both blood stage and sporozoite parasites [4]. Similarly, a combination of low doses of interleukin 12 and chloroquine induced mice immunity against reinfection with *Plasmodium chabaudi *[5]. The use of chloroquine in immunization procedures in humans has also been tested in a proof-of-concept clinical study [6], which showed that chloroquine-treated volunteers achieve higher levels of protection after inoculation with very small doses of intact sporozoites as compared to vaccination procedures based in the inoculation of radiation-attenuated sporozoites [7]. A plausible explanation of such behaviour is that the exposure to the pre-erythrocytic stages of infection, which is not avoided by chloroquine treatment, may enhance the immune response by favouring the presentation of antigens [8]. Anti-malarial drugs showing intra-erythrocytic parasitostatic effect might produce the immunization of the host by a similar mechanism, in which a controlled parasitaemia level would enhance antigen presentation to the immune system.

Maslinic acid (MA), a natural pentacyclic triterpenoid found in the olive fruits, has been recently shown to display a parasitostatic effect on the intraerythrocytic cycle of *in vitro *cultured *Plasmodium falciparum *[9]. The anti-malarial activity was dose-dependent, showing parasitocidal effect at 100 μM, while a non-permanent arrest of parasite maturation in infected erythrocytes was observed at 30 μM. The parasite development from early ring to late trophozoite was halted as long as MA was present in the culture, and growth was resumed upon removal of the triterpene. As a natural product present in the human diet, MA can be regarded as safe in therapeutic applications. In addition, it has been shown that MA displays low toxicity on non-tumoral cells [10,11]. MA can be readily obtained in large quantities from olive pomace oil, a byproduct of olive processing, fulfilling thus important requirements for its eventual clinical application.

While parasitostatic drugs may not completely avoid the infective process like other currently used compounds, they might eventually mimic the effect of vaccination with inactivated parasite, as the presence of growth-arrested infected erythrocytes would trigger an enhanced immune response by the host.

In the present study, the protection exerted by MA against the *P. yoelii *17XL lethal strain in a model mice system was investigated. The possible effect of MA on the development of protective acquired immunity to malaria was also tested, and its use as a model for the development of parasitostatic anti-malarial drugs is discussed.

## Methods

### Parasites and animals

The rodent malaria parasite *P. yoelii *17XL (MRA-267) was obtained from the Malaria Research and Reference Resource Center, maintained by serial blood passage in mice and the infected blood was stored at -80°C. Random-bred ICR female mice (Hsd:ICR[CD-1^®^]) 6-8 weeks old, were purchased from Harlan Laboratories. The mice were housed under standard conditions of light and temperature in the Animal Housing Facility at Complutense University. All mice were fed *ad libitum *on a commercial diet. *In vivo *experiments were carried out in accordance with national and international guidelines for animal care, using protocols approved by the Animal Experimentation Committee of the Complutense University.

### Assay for *in vivo *anti-malarial activity

Maslinic acid (MA) was kindly provided by Dr. Andrés García-Granados from the University of Granada (Spain). The stock solution (200 mM) was prepared in 100% dimethyl sulfoxide (DMSO).

The *in vivo *anti-malarial activity of MA was analysed by using a four-day-blood suppressive test as previously described [12]. Briefly, mice were inoculated by intraperitoneal injection (i.p) of 10^7 ^red blood cells from *P. yoelii *-infected mice. The chemotherapy treatment started 2 h later (day 1) with a single dose of MA by a intraperitoneal injection of MA (40 mg kg^-1 ^day^-1^) followed by identical dose administration for the following 3 days. The control groups received DMSO 5% in phosphate buffered saline solution. The parasitaemia was monitored daily by microscopic examination of Wright's-stained thin blood smears.

To assess protective immunity, cured mice were challenged with the same dose of parasites 40 days after recovery from the primary infection and parasitaemia was further monitored for 30 days.

### IgG and IgM concentration in mice serum

*Plasmodium yoelii *specific antibodies present in sera from challenged mice were quantified by mouse-IgG and mouse-IgM ELISA kits (Bethyl Laboratories, Inc). 96-well plates were coated with 100 μL/well of each standard or sample, covered and incubated at room temperature (20-25°C) for 1 hour. After several washes, the wells were incubated for 1 hour with 100 μL of the primary anti-IgG or anti-IgM antibodies followed by 100 μL of enzyme-labelled secondary antibody for 30 minutes. The enzymatic reaction was developed following the manufacturer instructions, the absorbance of the reaction product was recorded in a microplate reader at 450 nm and immunoglobulin concentrations were calculated from standard curves.

### Western blot analysis

The specificity of immunoglobulins against *P. yoelii *proteins was further ascertained by immunoblotting. The parasite antigens were obtained from *P. yoelii *infected erythrocytes (iRBC) collected from mice showing 50% parasitaemia. The lysates were prepared with saponin in PBS (final concentration 0.1% w/v) by disrupting the erythrocyte and parasite vacuole membranes. The released parasites were washed with ice-cold PBS. *P. yoelii *proteins were then solubilized in 50 mM Tris-HCl pH 8, 50 mM NaCl, 3% CHAPS, 0.5% MEGA10, and gently mixed at 4°C for 15 min followed by three freeze-thaw cycles. The supernatants obtained after centrifugation (13,000 g, 10 min 4°C) were collected and referred to as parasite extracts. The protein concentration of the extracts was determined using a Bradford Reagent from Sigma-Aldrich. 10 μg of parasite proteins were fractionated on 12% SDS-PAGE (Bio-Rad) and transferred onto PVDF (Hybond-P, GE healthcare) membranes following standard procedures. Blots were incubated with mice sera antibodies for 2 h at room temperature, followed by HRP conjugated anti-mouse IgG (Amersham Biosciences) at 1/5,000 dilution. Detection was performed using the SuperSignal chemiluminescent substrate (Pierce) and exposure to X-ray film.

### Statistical analysis

The statistical analysis of data was performed using SigmaPlot11 software. Survival data was analysed using the Kaplan-Meier statistical method with Log-Rank significance test. Statistical significance (*P *value) of parasitic load was ascertained by performing t tests. *P *values < 0.05 were considered statistically significant.

## Results

### Effects of maslinic acid on a *P. yoelii *ICR mice model

**Figure 1:**
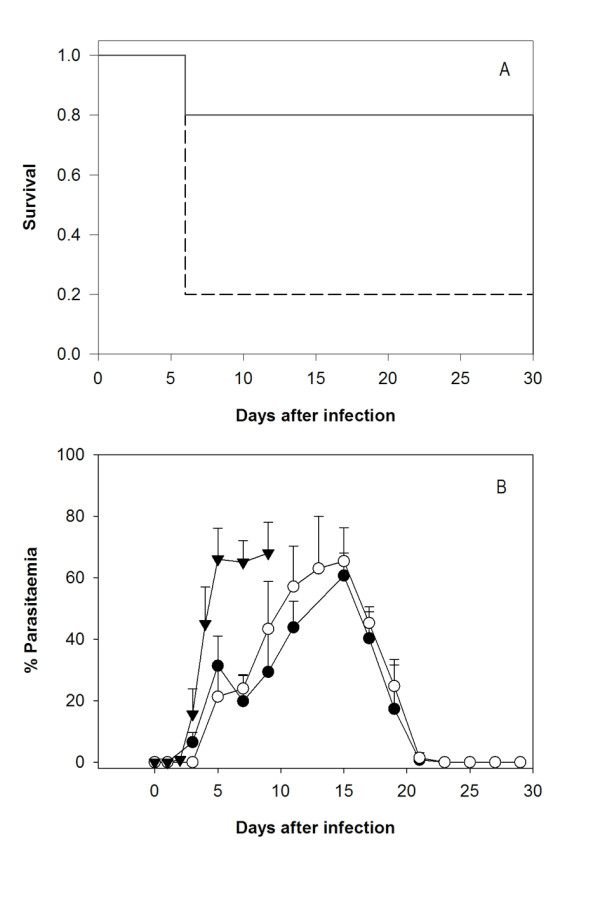
**Survival and parasitemia evolution after MA treatment**. ICR mice were infected with 10^7 ^*P. yoelii *17XL infected RBC and treated 2 hours post-infection with maslinic acid 40 mg Kg^-1 ^day^-1^, (0.8 mg per mouse per day) for 4 days, starting 2 hours after infection. A) Survival of treated mice (solid line, n = 10) and untreated control (dashed line, n = 10). Results are presented as the survival percent of one representative experiment. *P *= 0.009 (statistically significant difference between the treated group and the control)**; **B) Parasitemia dynamics in MA-treated (n = 10) and non-treated (n = 10) groups. Black circles: Survivors from MA-treated group; white circles: survivors from non-treated group, and triangles: non-surviving mice. †: 100% mortality. Results are presented as mean+SD of one representative experiment. Day 7, P < 0.001 survivors (treated or untreated) *vs *non-survivors.

In a previous report, a parasitostatic effect of MA on *P. falciparum*-infected erythrocyte cultures was evidenced by growth arrest along the ring-trophozoite stages [9]. To assess its possible anti-malarial activity *in vivo*, outbred ICR mice were treated by intraperitoneal administration of MA at 40 mg Kg^-1 ^following a 4-day suppressive test protocol. Both survival and parasitaemia evolution were monitored daily. As shown in the Figure 1, nearly 20% of ICR mice could overcome primary infection without any experimental intervention. These surviving mice developed peak parasitaemia levels of about 60% at day 15, and resolved their infections five days later. In comparison, the mice which could not overcome the infection achieved 60% parasitaemia at day 6-7 and none of them survived beyond day 10 (figure 1B). This result suggests the presence of a naturally resistant subpopulation to *P. yoelii *in the ICR strain.

Remarkably, the survival rate of ICR mice was significantly increased to 80% in the groups treated with MA, demonstrating the anti-malarial activity of this compound *in vivo*. Both treated and non-treated ICR mice which ultimately survived the infection exhibited similar parasitaemia profiles, showing slower increments throughout the first five days after infection as compared to the parasitaemia of non-surviving mice. The number of infected erythrocytes in these mice declined steadily from day 15, and at day 20 no parasites could be detected microscopically.

**Figure 2:**
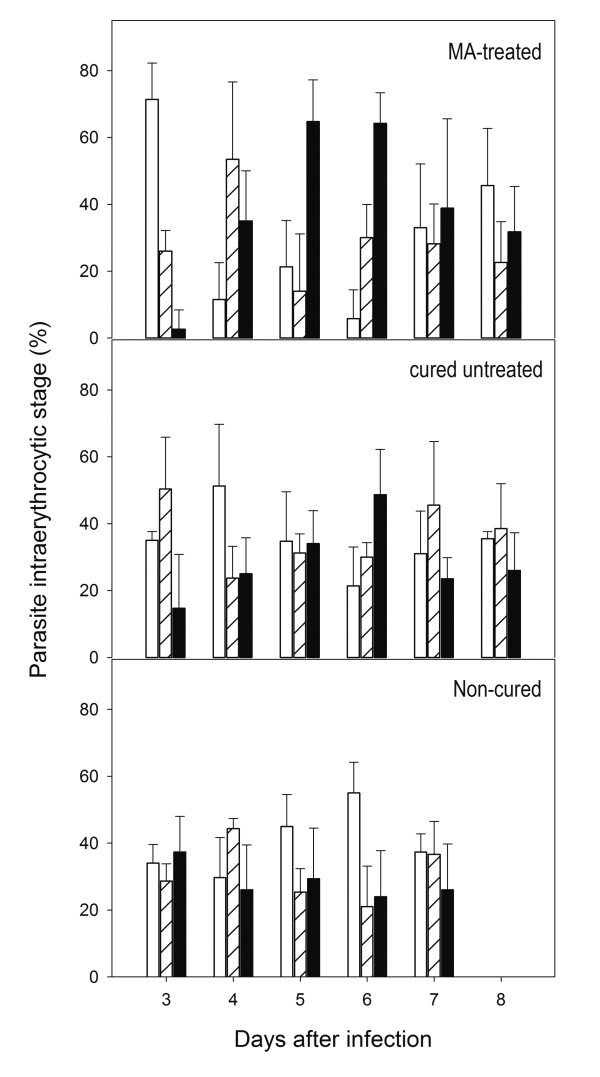
**Distribution of intraerythrocytic parasite stages in ICR mice**. Parasitic stages observed in blood samples from infected ICR mice. The fraction of ring- (white), trophozoite (dashed) and schizont-stage (black) iRBC from day 3 to 8 after infection is shown for non-surviving, non-treated surviving and MA-treated surviving mice. The data were obtained by microscopic inspection of Wright´s-stained thin blood smears and compared with controls without drug. Results are presented as mean+SD of five mice, each one accounting the percent of cells showing every parasite stage from a total of 1000 erythrocytes.

The comparison of parasitaemia profiles of cured *vs*. not cured mice displayed additional differences. In addition to lower multiplication rates, the parasite growth was halted in surviving mice between days 3 and 8 (Figure 1B). At concentrations corresponding to the IC_50_, MA displays a parasitostatic action on *P. falciparum*-infected erythrocytes [9]. To ascertain if MA can also delay the parasite growth *in vivo*, the distribution of parasite stages in erythrocytes from day 3 to 8 were analysed microscopically. As shown in Figure 2, parasites in MA-treated mice showed a synchronized stage distribution, as compared to the characteristic lack of synchrony known for *P. yoelii *infections, which was observed in non-survivors. At day 3, ring-stage was the predominant form, while trophozoites accumulated at day 4 and schizonts at days 5 and 6. Not-treated survivor mice showed a much less evident divergence in the frequency of schizonts at days 3 to 6, with no significant differences in ring and trophozoite-stages, suggesting that the mechanisms leading to growth delay in the non-treated and MA-treated mice during the first days of infection may not be identical. Compared to the 22-25 hours known to be required to complete a *P. yoelii *infective cycle in the erythrocyte [13], MA-treated mice infected erythrocytes required nearly 72 hours to reach schizogony from ring stage, revealing a strong interference of this compound with the parasite maturation.

### Parasitaemia course after rechallenge with *P. yoelii*

**Figure 3:**
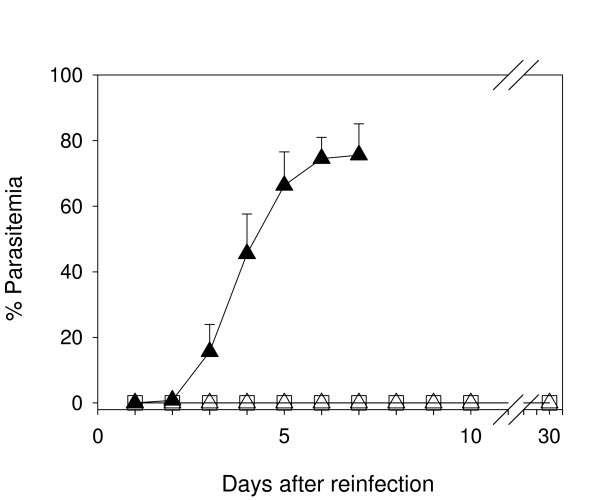
**Parasitemia in reinfected ICR mice**. Mice surviving primary infection from the MA treated (n = 8) and non-treated (n = 8) groups were reinfected at day 40 with 10^7 ^*P. yoelii *17XL, and the parasitemia was monitored daily for 30 days. White squares: MA-treated group reinfected; white triangles: untreated group reinfected; black triangles: naïve mice infected. †: 100% mortality. Results are presented as mean+SD of one representative experiment

To ascertain if mice cured from primary infection remained protected against subsequent plasmodium inoculations, survivors from both MA-treated and untreated subsets were rechallenged at day 40 (18 days after parasitaemia clearance) with a *P. yoelii *inoculum equivalent to the first infection. Parasitaemias were monitored for 30 additional days. As shown in Figure 3, no parasites could be detected microscopically in any of the reinfected mice. All those animals survived, showing neither malaria symptoms nor detectable parasitaemia, indicating that they were fully protected against *P. yoelii *17XL reinfections, likely by an acquired sterilizing immunity.

### Humoral response in mice after first infection and rechallenge

**Figure 4:**
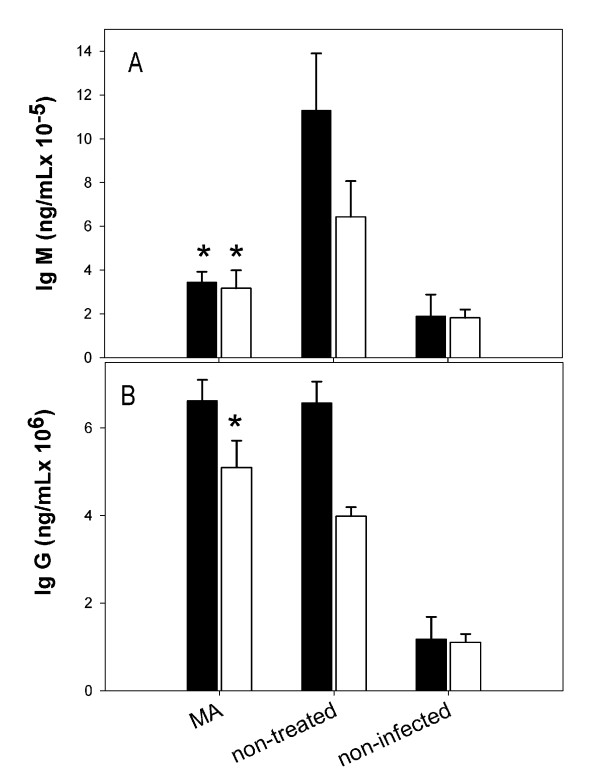
**Parasite-specific IgG and IgM antibody responses of ICR mice after *P. yoelii* infection**. Mice were infected with 10^7 ^*P. yoelii *17XL and treated with maslinic acid or left untreated. Sera from cured mice of the primary infection were collected at day 30. Sera from not cured mice correspond to day 5 after first infection (black bars). The surviving mice were reinfected at day 40 and sera was collected at day 10 after the reinfection (white bars). Results are presented as mean+SD immunoglobulin concentrations from three measurements, each containing pooled sera obtained from 6 mice. Statistical significant differences (*P*< 0.05) between infected MA-treated and non-treated groups are indicated by an asterisk.

To gain some insight on the development of immune response against *P. yoelii *both after primary infections and rechallenge, the concentrations of IgG and IgM immunoglobulins in sera were determined by ELISA. The results, shown in Figure 4, revealed that while IgM concentration remained relatively high in non-treated mice 30 after first infection, lower concentrations were detected in MA-treated mice. Furthermore, such difference in IgM levels was also observed in sera collected 10 days after rechallenge, suggesting a more effective acquired immune response in MA-treated mice. On the contrary, IgG values were equivalent in MA-treated and non-treated mice, showing instead lower concentrations in sera from rechallenged mice as compared to the primary infection. This reduction may be a consequence of low induction IgG synthesis due to the minute amount of parasites in blood, as no newly infected erythrocytes became apparent after the reinfection.

**Figure 5:**
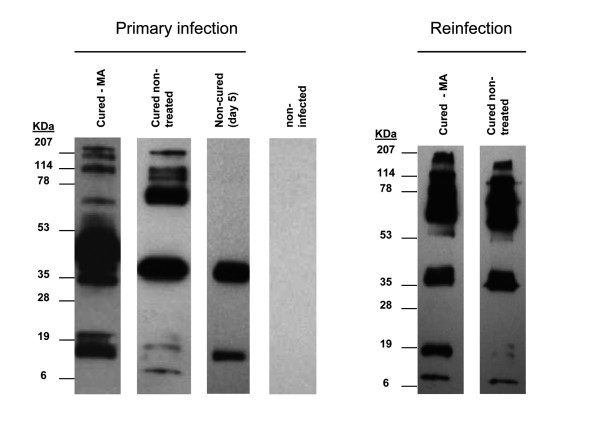
**Immunoelectrophoretic analysis of anti-*P.yoelii *immunoglobulins present in sera from cured and non-cured ICR mice**. Total protein extracts (10 μg) from Py17XL were separated in 12% gradient PAGE-SDS, transferred to PVDF membranes and blotted with pooled sera from six ICR mice for each indicated infection condition. Reinfection details are indicated in figure 3. Immunoglobulins were visualized using anti-mouse IgG-HRP.

To get further information on the specific humoral response, Western-blot analysis of *P. yoelii *proteins incubated with sera from both treated and non-treated mice and anti-mouse IgG-HRP were performed. Sera from surviving mice were collected at day 30 after first infection, when no parasites could be detected in blood smears. The profile of proteins immunodetected with sera from mice which survived the first infection revealed the presence of a wide range of antibodies reacting with *P. yoelii *antigens (Figure 5). No reacting antibodies could be seen in uninfected mice. A strong antigenic band at approximately 45 kDa, and secondary bands at the 8-20 kDa and 60-200 kDa ranges were observed in treated mice. Sera from non-treated surviving mice showed some different pattern, with the strongest signals at 40 kDa and 70 kDa, indicating that the humoral response in MA treated mice may not be identical to that of the naturally resistant mice. Sera from infected mice which do not survive the infection was collected at day 5, showing two reacting bands at 40 and 20 kDa. This suggests that antibodies binding to these two antigens are probably the first to be synthesized, but either they are not able to provide protection by themselves against the infection, or they are delivered too late when the rate of parasite growth has reached a point of no-return.

The immunoreactive protein pattern in mice after reinfection was also analysed by western-blot of sera collected at day 10 after the second challenge (Figure 5). Compared to the blots from the first infection, a large increase in the signals obtained at high molecular weight proteins (60-200 kDa) was observed. In addition, the band at 8 kDa also yields a strong signal in all sera from cured mice. These results could be explained assuming that antibodies reacting with those high molecular proteins, and/or the 8 kDa peptide, are produced along the high-parasitaemia episode in the first infection, leading to the elimination of the parasite after day 15. Upon reinfection, the same set of antibodies would be massively synthesized, avoiding even the onset of the infection.

## Discussion

Research efforts on the discovery of new anti-malarial drugs has generated only a handful of successful compounds suitable for the treatment and prevention of acute symptoms of malaria [14], which are not fully effective for the overall control of the disease in endemic areas. The search of useful vaccines also remains elusive despite the many breakthroughs in the molecular biology, genetics and immunology of *Plasmodium *infections. An alternative to these two approaches may lay in the development of drugs which, while keeping under control the development of the parasite, would allow the host immune system to generate a strong response leading to parasite clearance and, eventually, leave the host immunized against further reinfections [15,16].

It was previously reported that maslinic acid inhibits the intraerythrocytic development of *P. falciparum in vitro *at ring-trophozoite stages, but does not kill the parasite, as the removal of the drug allows the infection to progress [9]. As shown in this report the infection outcome of *P. yoelii*-infected mice is also affected by MA.

About one-fifth of outbred ICR population showed to be naturally able to overcome the acute phase, reduce parasitaemia to undetectable levels and get cured after infection with the lethal strain of *P. yoelii*. Such parasitaemia dynamics, in which the density of infected RBC increases progressively to reach a peak and then declines, resembles the dynamics of plasmodial infections in humans, where the majority of infections, even by lethal *P. falciparum *strains, course with an acute phase which is followed by reduction of parasitaemia levels, to remain as chronic or asymptomatic [1,3]. The parasitaemia in non-cured ICR mice showed a four-fold increase in 48 h, while cured ICR displayed a slower rate, with a doubling time of 4 days. A closer inspection of the parasitaemia increase in surviving mice during the first days of infection reveals that the multiplication of the parasite is practically blocked between days 5 and 11, maintaining during this period a steady parasitaemia below 30%. After this episode the parasite takes 4 days to double its blood concentration and then, by day 15, progressively decay until undetectable levels at day 20. These results are coherent with previous observations with non-lethal strains of *P. yoelii *[17] and *P. chabaudi *[1], where CD4^+^T helper 1 cells, interferon-γ [18] and proinflamatory cytokine interleukin-12 [19] have been shown to be required for the control the parasitaemia within the first 7 to 14 days after infection. The survival of some 5% of inbred BALB/c mice to lethal *P. yoelii nigeriensis *has also been reported [20], showing low parasitaemia peaks (5%) at day 6. In the same report it was shown that a mixture of B and T cells from surviving mice was able to provide protection to an X-radiated, naïve recipient. It has been proposed that the innate immune mechanism is essential to limit the initial phase of parasite replication and allowing the host time to develop antibody-mediated specific adaptative responses enabling clearance of infection [1]. The presence of a minor fraction of ICR mice population able to trigger this innate response in time to avoid the lethal growth of the parasite may explain the surviving of 20% of ICR mice.

A four-day MA treatment with a dose of 40 mg Kg^-1 ^day^-1 ^after Py7XL infection led to an increase in survival from 20% to 80% of mice, with a parasitaemia profile similar to that of the naturally surviving mice. The simplest way to explain the increase in surviving mice associated to MA treatment would be to assume that all ICR mice harbour the required elements to trigger this innate immune response, but the time window for this response to be effective is short, in such a way that, in the majority of the infections of ICR, the parasite fast growth overcomes the immune response. The parasitostatic effect of MA may take place at this decisive point, by delaying the maturation of plasmodium at early stages and providing the system extra time to deploy the primary response. This is further supported by the observed parasitostatic effect of MA *in vivo*, which leads to the synchronization of Py17XL growth stages in the first 6 days of infection. As the drug was administered from day 1 to 4, it is conceivable that newly infected RBC remained at ring-trophozoite stages during days 3 and 4, starting to reach schizont stage when the drug concentration in blood decays after treatment. Such growth hindering would increase the fraction of mice able to control the parasitaemia and survive. Remarkably, no parasite synchronization was observed in non-cured mice in the same time-period. Cured non-treated mice showed some decrease in the frequency of schizonts at day 3 followed by a progressive increase up to day 6, while no apparent differences were observed in ring and trophozoite distributions. This suggests that the nonspecific early immune response in the naturally resistant subpopulation of ICR may be affecting primarily the parasite egress from the schizonts rather than delaying the maturation at earlier stages.

All ICR mice which survived the primary infection, independently of treatment, also remained immunized against rechallenge. This protection prevents any intraerythrocytic growth of the parasite, as no parasitemia was detected even 30 days after reinfection. Earlier work on ICR infected with avirulent *P. yoelii *revealed the induction of long-term immunity against virulent *P. yoelii *and *Plasmodium berghei *[21]. The present results extend those findings to subpopulations of naturally resistant ICR mice infected with virulent *Plasmodium*, supporting the validity of the observed pattern of immune response against *P. yoelii *in mice. Similarly, it has been shown that chloroquine or artesunate-treated both resistant DBA/2 infected with lethal Py17XL, or non-resistant BALB/c infected with non-lethal Py17XNL, remain immunized against homologous challenge [22]. These experiments showed no reduction in rates of parasite appearance after reinfection as compared to untreated control groups. Furthermore, no significant differences were observed in that study in IFN-γ or parasite-specific IgG levels in serum. As chloroquine and artesunate reduce drastically the parasitaemia during the first infection, it is likely that this would also reduce the interaction of the immune system with parasitized cells, precluding the heighten of immune response detected in MA-treated mice.

Remarkably, other experiments carried out using BALB/c mice infected with lethal *P. yoelii *[20] showed that survivor mice remained fully susceptible to re-challenge infections. This apparent discrepancy with ICR may be explained considering the low parasitaemia peaks observed in BALB/c mice (5%) as compared to ICR (60%). Thus, the lower exposure to parasite antigens in BALB/c may not be sufficient to develop a long-term immune memory. Accordingly with this possibility, low doses of parasitocidal drugs, which would allow the development of parasitaemia during first infection, may increase the protection against reinfection, as it has been reported for chloroquine-treated mice infected with *P. berghei *[15] and a combination therapy consisting of low doses of interleukin 12 and chloroquine [5].

It is currently accepted that specific adaptative immunity, dependent on B, CD4^+ ^T and NK cells, and IFN-γ, is required for parasite clearance once the innate non-specific response manages to control parasitaemia during the acute phase in mice [1,23]. The cellular and humoral response developed at the end of the acute phase in cured ICR mice may thus provide protection against further reinfections. IgM levels in serum are significantly lower in MA treate mice. As no parasitaemia could be detected at day 30, the high levels of IgM surviving untreated mice may denote the presence of sub-microscopic levels of parasites blood. The relatively low levels of IgM in treated mice as compared to untreated suggests that, in spite of the similar parasitaemia profiles in all survivors, the parasite clearance may be more efficient in mice treated with MA. Alternativelly, the differences in IgM levels may be a consequence of the seletion exerted by the infection. Thus, mice producing consistently higher levels of relevant IgM may be those that survive even without treatment, while individuals producing low levels of IgM would survive only when treated with MA. In any case, MA treatment would be determinant to increase survival.

The antigenic profiles observed after the primary and second infections reveal the presence of a wide variety specific antibodies against *P. yoelii *antigens. Furthermore, the comparison of the immunogenic protein profiles from cured and not cured mice may allow the identification of potential protective antigens: thus, both high- and low-molecular weight proteins appear to be highly antigenic in samples of cured mice 30 days after the first infection, and antibodies recognizing them are even incremented in the immunized mice after the reinfection. The identification of these antigens may provide further insights in the analysis of the protective immune response against malaria.

In human malarial infections asexual intraerythrocytic stages can induce sterilizing immunity [24-26]. It has been observed as well that experimental exposure to the parasite can induce in humans an improved sterilizing immune response when anti-malarial drug treatment is co-administered [6]. Moreover, repeated sub-clinical infections following sporadic drug treatment also facilitate the development of immunity to malaria [27]. Thus, the use of parasitostatic compounds which, like maslinic acid, enhance the host probability to develop effective innate and adaptative immunities is worth to be explored. The characteristics of the acquired immunity produced by this kind of treatment should be analysed in terms of long-term protection, cross-immunity and the effects on non-erythrocytic plasmodial stages.

The elucidation of the targets of maslinic acid would also allow the selection of a new class of specific drugs aimed to facilitate the host immune-response. Such drugs may also be advantageous, as the low selective pressure exerted on the parasite would prevent the emergence of resistant variants.

## Conclusion

A subpopulation of random-bred ICR mice may develop natural resistance to *P. yoelii *infection during the first days after infection, leading to a slower rate of parasite growth which, in turn, would facilititate an efficient host response to infection and parasite clearance. Maslinic acid appears to enhance the chances to trigger this response in the treated mice, increasing the fraction of individuals which can control the parasitaemia and survive the first infection. Surviving mice from the primary infection remain protected against reinfection, showing an enhanced variety of antibodies against *P. yoelii *antigens.

## Competing interests

The use of maslinic acid as antiparasitic agent is protected by a patent owned by the University of Granada (date of filing: March 29, 2007; Patent Number: WO/2007/034009). The authors declare no competing financial interests.

## Authors' contributions

CM and PM carried out the laboratory work, contributed in the analysis of data and helped to draft the manuscript. JMB, AD and AP participated in the analysis and interpretation of the data, and wrote the manuscript. AD and AP conceived and coordinated the study. All authors read and approved the final manuscript.
